# Mitochondrial Rejuvenation After Induced Pluripotency

**DOI:** 10.1371/journal.pone.0014095

**Published:** 2010-11-23

**Authors:** Steven T. Suhr, Eun Ah Chang, Jonathan Tjong, Nathan Alcasid, Guy A. Perkins, Marcelo D. Goissis, Mark H. Ellisman, Gloria I. Perez, Jose B. Cibelli

**Affiliations:** Cellular Reprogramming Laboratory, Michigan State University, East Lansing, Michigan, United States of America; University of Colorado, Boulder, United States of America

## Abstract

**Background:**

As stem cells of the early embryo mature and differentiate into all tissues, the mitochondrial complement undergoes dramatic functional improvement. Mitochondrial activity is low to minimize generation of DNA-damaging reactive oxygen species during pre-implantation development and increases following implantation and differentiation to meet higher metabolic demands. It has recently been reported that when the stem cell type known as induced pluripotent stem cells (IPSCs) are re-differentiated for several weeks *in vitro*, the mitochondrial complement progressively re-acquires properties approximating input fibroblasts, suggesting that despite the observation that IPSC conversion “resets” some parameters of cellular aging such as telomere length, it may have little impact on other age-affected cellular systems such as mitochondria in IPSC-derived cells.

**Methodology/Principal Findings:**

We have examined the properties of mitochondria in two fibroblast lines, corresponding IPSCs, and fibroblasts re-derived from IPSCs using biochemical methods and electron microscopy, and found a dramatic improvement in the quality and function of the mitochondrial complement of the re-derived fibroblasts compared to input fibroblasts. This observation likely stems from two aspects of our experimental design: 1) that the input cell lines used were of advanced cellular age and contained an inefficient mitochondrial complement, and 2) the re-derived fibroblasts were produced using an extensive differentiation regimen that may more closely mimic the degree of growth and maturation found in a developing mammal.

**Conclusions/Significance:**

These results — coupled with earlier data from our laboratory — suggest that IPSC conversion not only resets the “biological clock”, but can also rejuvenate the energetic capacity of derived cells.

## Introduction

Induced pluripotent stem cells (IPSCs) are emerging as an important new tool to model human disease with potential to be used one day in the treatment of these same disorders. With regard to cell replacement, IPSCs have the distinct advantage that they can be derived from somatic cells such as skin fibroblasts from essentially any donor subject for reintroduction to that same individual as an autologous transplant. They are advantageous for the study of human disease because they are essentially “pre-validated” and may be derived directly from individuals with clinically confirmed illness[1], [2].

Multiple studies have determined that IPSCs are very similar to ESCs in their pluripotency and capacity to differentiate[3]–[5], and more recent studies have sought to determine whether IPSCs and their derivatives also show signs of cellular rejuvenation following adoption of the ESC-like phenotype[6]–[10]. We have recently described a matched set of cell lines derived from two fibroblast (FIB) lines — FIBA and FIBB – designed to answer questions regarding the “before and after” specifics of IPSC conversion[7]. Both FIBA and FIBB display average telomere lengths that are approximately equivalent (10.25 Kb and 10.76 Kb respectively) even though both lines were derived from individuals of very different ages (EW16 and 70 yrs, respectively). This is likely due to the relatively high growth rate and number of population doublings that rendered FIBA comparable in cellular age to the lower-passage and more recently-derived FIBB from an elderly donor. Despite the fact that both lines were relatively old in cellular terms, both yielded multiple IPSC lines displaying all of the hallmarks of human ESCs, and displayed an average telomere elongation of >40% (10.5 Kb to 15.4 Kb) compared to input cells[7]. Together, these results suggested that reprogramming restored at least one key indicator of cellular age – telomere length – following adoption of the IPSC phenotype. In addition, unlike mouse IPSCs where telomere elongation rivaling mESCs was not observed until approximately passage 30[6], average human IPSC telomere lengths equaled or exceeded average hESC telomere lengths (15.4 Kb vs 13.9 Kb) by passage 5, the earliest time-point examined. Recently, other groups have reported similar increases in IPSC telomeres derived from other fibroblast lines[10].

Of equal importance to the observation that reprogramming to pluripotency leads to telomere elongation, we also found that after differentiation and multiple population doublings, telomere shortening was resumed in IPSC-derived cells. Since telomeres only shorten at a rate of approximately 50–100 bp/replicative cycle[11]–[14], in order to observe measurable telomere decreases in IPSC-derived cells, we elected to establish and analyze fibroblast lines derived from IPSC teratomas to better mimic the degree of growth and differentiation that may be incurred during normal human development. In the majority of teratoma-derived fibroblast lines (referred to as TER lines), the combined number of replicative cycles required for teratoma growth and the subsequent production and expansion of homogenous TER lines produced a dramatic loss of telomere length (from an IPSC average of 15.4 Kb to a TER average of 10.4 Kb)[7]. This result, coupled with morphologic observations, immunocytochemical indicators, and genome-wide methylation analysis confirmed that TER cells were differentiated fibroblastic cells that had resumed normal cellular aging following loss of pluripotency. While this result suggested that IPSC-derived cells regained the capacity for many population doublings as the result of conversion to an ESC-like state (50–100 added doublings, assuming that each kilobase of telomere translates into 10–20 added cycles), these results could give no indications regarding the overall robustness or “energetics” of the derived cells. A better understanding of the rejuvenative state of IPSC-derived cells is important for at least two reasons. First, if after the derivation process, the resulting cells have substantially longer life but retain the cellular properties of a relatively old donor cell, then donor age is an important consideration in future cell replacement therapies, whereas, if IPSC-derived cells not only can live longer, but also take on the robust energetic properties of youthful cells, cellular replacement from donors of any age would be predicted to have equal positive potential.

The second reason that an understanding of the functional “age” of IPSC-derived cells is important relates to the burgeoning use of IPSCs to study human disease. Results from our laboratory and others suggest that the quality and cellular age (as measured by telomere length) of IPSCs derived from young and old subjects are essentially equivalent; however, as more and more differentiated cell models derived from donor subjects of disparate ages become available, it becomes important to know whether, and to what extent, observed differences may be influenced by donor age. If IPSC-derived cells are capable of more cell divisions, but in other respects “behave” like cells of the donor age, this suggests that age-matching will be critical to establishment of useful model cell lines, whereas if IPSC conversion rejuvenates both cell cycle capacity *and* other cellular properties, then cross-comparisons of IPSC-derived cultures without concern for donor age would be reasonable, but the use of IPSC-derived cultures from elderly donors with the expectation of modeling “aged” cell types would not be valid in most instances.

One measurement of cellular health and energetics that correlates with both cellular and organismal age is mitochondrial function[15]–[17]. In mammals, all mitochondria are maternally derived from the oocyte. A high-quality oocyte mitochondrial complement is critical to provide the best possible start for the generation of a completely new individual[18]. While oocyte mitochondria are robust, shortly after fertilization the mitochondrial complement is diluted by cell division and becomes much less active, in part because of the low-oxygen environment without direct access to the blood supply, but also because mitochondrial activity and oxidative phosphorylation (OXPHOS) create reactive oxygen species (ROS) as a by-product that can damage DNA and introduce mutations. DNA damage at the very earliest stages of development giving rise to even small changes to the genome could have catastrophic consequences as dividing cells are propagated throughout the developing organism, hence, the mitochondrial complement of ESCs *in situ* is both small and of low activity[18]–[20].

With the establishment of differentiation in the developing embryo, the mitochondrial complement both diversifies and amplifies depending on the cell type. Typically, somatic cell types such as newly-derived embryonic fibroblasts have efficient mitochondrial complements and abundant ATP, and as they age (either *in situ* or *in vitro*), accumulate larger proportions of mitochondria in increasingly active configurations in an effort to keep up with energetic demands. Cell types such as CNS neurons, skeletal, and heart muscle adopt complex mitochondria configured for maximal respiration and ATP production. The impact of age on mitochondrial function in these tissues is evidenced by the progressive nature of neuropathies and myopathies in patients with mitochondria compromised by disease [21].

To better understand the relationship between cellular identity, cellular age, and their impact on mitochondria, two recent reports have compared several properties of the mitochondrial complement of input fibroblasts, IPSCs, and IPSCs allowed to differentiate for several weeks in culture[22], [23]. Their results indicate that like ES cells, IPSCs display reduced ROS production that stems from a mitochondrial complement reduced in number and mass from the level in input cells to a level similar to hESCs. Analysis of differentiating IPSC cultures revealed that, for the most part, mitochondrial properties returned to the pre-reprogrammed state similar to input fibroblasts following several weeks of differentation.

Together, these results suggested that although the IPSC conversion process resets the biological clock, other important measures of cell fitness such as mitochondrial function, is either not improved or is even slightly compromised compared to input cells.

We have analyzed the structural and functional properties of mitochondria in the FIB, IPSC, TER, and ESC lines characterized for telomere elongation and found substantial differences in the TER mitochondrial complement derived after long-term growth and differentiation. Our results confirm that IPSCs display mitochondria very similar to their natural ESC counterparts, but that after redifferentiation the mitochondrial complement has undergone a dramatic functional improvement compared to both IPSCs and parental fibroblast lines. Our results indicate that IPSC conversion resets not only the *replicative* — but also the *energetic —* capacity of derived cells, suggesting that even tissues from elderly individuals can be restored to a biological age functionally equivalent to new-born cells.

## Results

**Figure 1:**
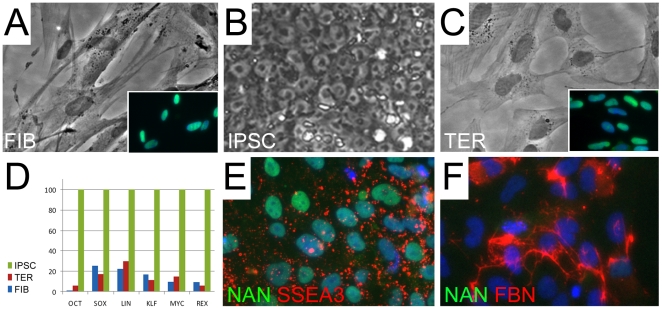
Morphological characteristics and marker expression of FIB, IPSC, and TER cells. FIBA and derivatives are used as examples. **A–C**) Phase-contrast images showing the morphology of FIB, IPSC, and TER lines as labeled. Insets in A and C show reactivity of FIBA and TERA with human-specific nuclear (HuNu) antigen antibody (green). Blue is DAPI co-stained DNA. **D**) Graphical representation of the relative expression of pluripotency-related gene mRNAs in all cell lines (IPSC value set at 100%). Pluripotency marker genes were detected at low levels in FIB and TER cells compared to IPSCs. **E–F**) Immunocytochemical analysis of IPSCs (**E**) and TER cells (**F**), indicating that TER cells had not only lost high-level of expression of pluripotency markers such as Nanog, but also gained expression of fibroblasts markers such as fibronectin (FBN) (see also Ref. 7). Magnification: 800X, insets 250X.

Mitochondria were analyzed in two human primary cell lines at three phenotypic stages: fibroblasts, IPSCs, and fibroblasts derived from IPSC teratomas. Two lines of human ESC were also analyzed for comparsion to IPSCs. The fibroblast cell lines — FIBA and FIBB — used in these analyses have been described previously[7], and were selected because at all stages of differentiation they appeared very similar to other cell lines used for analysis of pluripotency[3], [4]. Furthermore, despite the fact that both lines were derived from donors of disparate ages, they displayed signs of cellular aging with telomeres of similar lengths[7] as discussed in the Introduction. The IPSC lines A and B (derived from fibroblast lines A and B), also displayed very similar morphologies, marker expression, global methylation profiles, capacity for teratoma formation, and telomere elongation[7] and were used in this study between passages 5–8. The re-differentiated fibroblast-like lines (TER lines) were produced from explants of IPSCA and B teratomas, expanded in vitro, and selected over multiple passages for cells of fibroblast morphology. TER cells appear morphologically similar to FIB cells with a small nucleus/cytoplasm ratio and a flattened stellate shape distinctly different from IPSCs (Fig. 1A–C). In addition to morphology, TER cells also resembled the input fibroblasts lines and differed dramatically from IPSCs in methylation profile and telomere shortening [7], and in the expression of multiple markers by both qPCR and immunocytochemical analysis (Fig. 1D–E). In addition to indirect indicators — such as the functionality of human-specific reagents in methylation and qPCR analysis – the human origin of TER cells was further confirmed by positive immunostaining for human-specific nuclear antigen (Fig. 1.A,C). Although no IPSC-derived cell is likely to be phenotypically identical to the parental input cell, we believe that TER cells represent the best approximation possible.

For functional analyses, four mitochondrial properties were measured for each cell type: 1) total cellular ATP, 2) ADP/ATP ratio, 3) mitochondrial mass, and 4) mitochondrial membrane potential, to determine if general mitochondria performance changed with dedifferentiation and redifferentiation and if direct reprogramming to the IPSC phenotype produced mitochondria functionally similar to the mitochondrial complement of human ESCs as previously reported[22], [23]. Previous studies [7] and ongoing experiments revealed few, if any, differences among cell lines within a type, so to increase the confidence of our analysis given the limited number of subjects available, the data were analyzed using a least-squares ANOVA with cell line effects nested within cell type.

**Figure 2:**
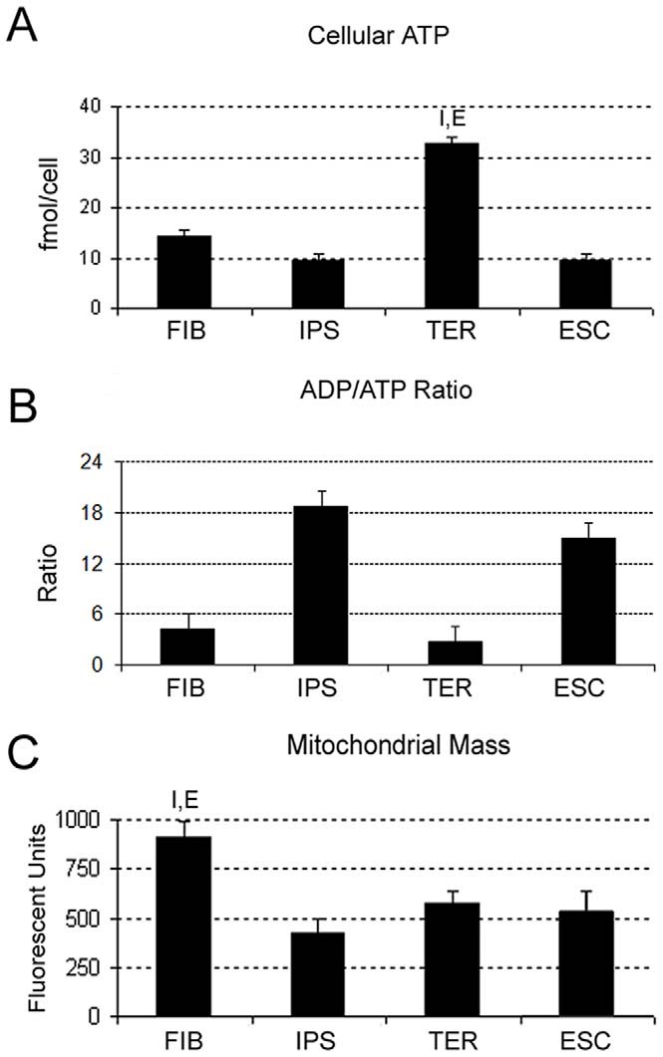
Functional comparison of mitochondria in FIB, IPSC, TER and ESCs. **A**) Total cellular ATP (fmoles/cell) in each cell type as labeled. **B**) ADP/ATP ratio in each cell type as labeled. **C**) Relative mitochondrial mass in tested cell types. Error bars indicate SEM. Letters above individual bars indicate values that differ statistically (P<0.05) from other cell type(s). (FIB = F, IPS = I, TER = T, ESC = E).

Measurement of cellular ATP was performed using a luciferase bioluminescence assay and the results are shown in Fig. 2A. Of the four cell types, TER cells displayed the highest cellular ATP content (32.84 fmol/cell) and differed significantly from both IPSCs and ESCs. IPSCs and ESCs displayed similar and low ATP levels (9.40 and 9.49 fmol/cell, respectively), while input FIB cells displayed ATP levels in the mid-range (14.21 fmol ATP/cell), closer to the pluripotent cell types than to TER cells.

Determination of the ADP/ATP ratio, a general indicator of cell metabolism, indicated that although TER cells displayed higher total cellular ATP than all of the other cell types, the relative ratio of ADP to ATP in TER cells was similar to the ADP/ATP ratio observed in parental fibroblasts (2.8 and 4.3, respectively) (Fig. 2.B). The ADP/ATP ratio for the differentiated cell types differed substantially from the ADP/ATP ratios in the pluripotent cell types (15.0 for ESCs and 18.8 for IPSCs).

The experiment shown in Fig. 2B suggested that overall metabolic activity as measured by the ADP/ATP ratio segregated along phenotypic lines; however, the overall higher ATP level seen in the TER cells in Fig. 2A remained unexplained. We reasoned that one explanation for this discrepancy might be a substantial difference in mitochondrial mass between TER cells and other cell types. Mitochondrial mass was assessed by NOA staining (Fig. 2C), and revealed that the mitochondrial mass of TER cells did not differ significantly from either IPSCs or ESCs. Only FIB cells displayed a significant difference in mitochondrial mass, with an approximate 2-fold increase over the other cell types. Far from explaining the elevated level of ATP in TER cells, indications that FIBs bore a larger mitochondrial complement than TER cells argued that FIB/TER differences were likely to reflect some form of improvement in mitochondrial quality as opposed to quantity.

**Figure 3:**
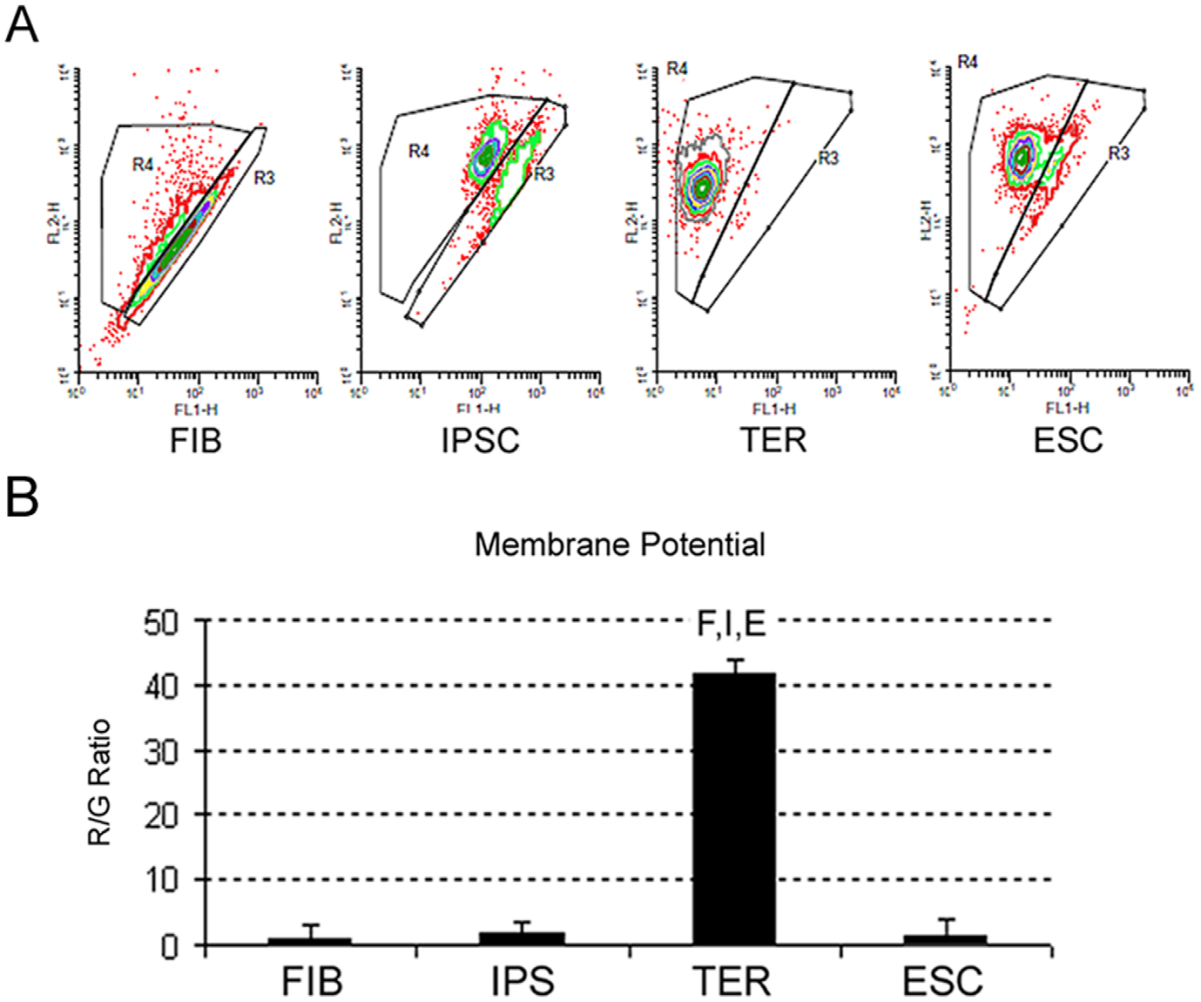
Analysis of mitochondrial membrane potential in FIB, IPSC, TER and ESCs. **A**) Representative scatter plots from flow cytometry analysis showing red/green JC1 dye fluorescence in cell types as labeled. The outlined area R4 is red fluorescence and area R3 is green fluorescence. **B**) Bar graph showing the ratio of JC-1 red and green (R/G) fluorescence indicative of mitochondrial membrane potential for each cell type. Error bars indicate SEM. Letters above individual bars indicate values that differ statistically (P<0.05) from other cell type(s). (FIB = F, IPS = I, TER = T, ESC = E).

Mitochondrial membrane potentials of all cell types were analyzed using flow cytometry and the vital dye JC-1[24] to further study the mitochondrial complement. These analyses shown in representative scatter plots in Figure 3A, indicate at a glance that TER cells displayed a dramatic and significant shift in the ratio of red-to-green differential fluorescence. Mitochondria with high membrane potential and a more active electron transport chain efficiently imported the JC-1 dye resulting in aggregation and red JC-1 fluorescence (delineated as region 4(R4) in the plots in Fig. 3A), whereas in less active mitochondria with lower membrane potential, the JC-1 remains in the monomeric, green-fluorescent conformation (region R3 in Fig. 3A). Quantification of the relative ratios are shown in Fig. 3B. Whereas FIB, IPSC, and ESC lines displayed R/G ratios between 1.1–1.9, the TER lines displayed an average R/G ratio of 41.8. This further indicated that the TER cell mitochondrial complement differed significantly from the input parental fibroblasts as well as the pluripotent cell types.

**Figure 4:**
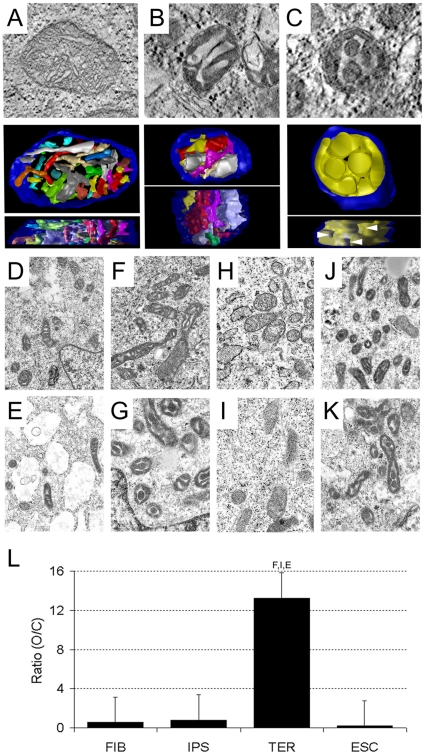
Structural comparison of mitochondria in FIB, IPS, TER, and ESC cell types. **A–C**) Electron tomography of the mitochondrial configurations observed. Three primary configurations were observed: **A**) *top panel*: A central slice through a tomographic volume showing the *orthodox* configuration. *bottom panel*: Top and side views of the segmented and surface-rendered volume of the orthodox mitochondrion showing individual cristae in various colors (top view (*upper*) side view (*lower*)). **B**) As in A, for the *condensed* configuration. **C**) As in A,B for the *ultracondensed* configuration. Arrowheads point to greatly enlarged crista junctions. **D–K**) Representative TEMI of mitochondria in all cell lines. (Mag = 7500-10000X). FIB and IPSC lines displayed a mix of mitochondrial configuration, most of condensed configuration (FIBA (**D**), FIBB (**E**), IPSCA (**F**), IPSCB (**G**)). TER cells displayed a preponderance of orthodox mitochondria (TERA (**H**), TERB (**I**)), and native ESCs displayed a preponderance of condensed mitochondrial forms (ESCA (**J**), ESCB (**K**)). **L**) Bar graph of the ratio of mitochondria scored as orthodox or condensed configuration (O/C) in FIB, IPS, TER, and ESC cell types as labeled. Error bars  =  SEM. Letters above TER bar indicate statistical difference (P<0.03) from all three other cell types.

To explore the underpinnings of increased TER cell mitochondrial efficiency, we employed electron microscopy and the advanced 3D technique of electron tomography (ET) to examine and quantify ultrastructural characteristics of the mitochondria in each cell type. Three-dimensional ultrastructural analysis can determine the overall disposition and complexity of individual mitochondrial crista and matrix – compartments formed by the inner mitochondrial membrane — that play a critical role in multiple mitochondrial functions including calcium storage, glutamate production, and most importantly, the efficient generation of ATP. ET was used to assess the complexity of mitochondrial architecture (Figure 4A–C). In the top panels of Figs. 4A–C, central slices through tomographic volumes of mitochondria illustrate the range of mitochondrial configurations observed in the cell lines analyzed. In the lower panels, reconstructions producing top- and side-views of surface-rendered volumes that facilitate visualization of the disposition and complexity of individual cristae (false colored) are shown. The upper panel of Figure 4A shows a mitochondrion with steady-state morphology known as the *orthodox* configuration, characterized in cross-section by tightly packed thin cristae filling an expanded, more translucent mitochondrial matrix. The reconstructions in the lower panels reveal a correspondingly complex cristae complement.

Figure 4B, on the other hand, shows a mitochondrial morphology referred to as the *condensed* configuration, and is characterized by mitochondria with a dark, condensed matrix and thus expanded, translucent cristae. Mitochondria can interconvert between the broadly defined orthodox and condensed configurations [25]. The complexity of cristae in mitochondria of the condensed configuration is manifest using a 3D technique such as ET. There are generally fewer and larger cristae in the condensed organelle, as displayed in the reconstructions in the lower panel of Fig. 4B, indicating that cristae remodeling needs to occur upon transition from the orthodox to the condensed conformation. An extreme of the condensed configuration is the *ultracondensed* configuration (Fig. 4C). The matrix of ultracondensed mitochondria appear as dense bodies, described as ‘sausages’ that snake through the mitochondrial interior, are often interconnected, and surround a single or small number of greatly expanded cristae where the crista junctions have been enlarged to several-times normal [26]. As shown in the tomographic reconstructions, ultracondensed mitochondria display complex topology of their crista architecture (Fig. 4C, lower panels).

Transmission electron microscope images (TEMI) from each cell type were examined to determine the morphological disposition of mitochondria. Representative images of FIB (Fig. 4D,E), IPSC (Fig. 4F,G), TER (Fig. 4H,I) and ESC lines (Fig. 4J,K) showed an apparent high degree of similarity among FIB, IPS, and ESC mitochondria, and a distinct difference in the morphology of TER mitochondria. To quantify these potential differences, mitochondria in a series of TEMI from each cell line were counted and assigned to one of two broad categories: orthodox or condensed.

The ratio of mitochondria in the orthodox configuration compared to condensed conformation is shown in Figure 4L. As suggested by the representative images of Fig. 4D–K, the FIB, IPS, and ESC lines displayed mitochondrial complements composed predominantly of mitochondria in a condensed configuration, whereas TER cells lines displayed an overwhelming preponderance of mitochondria in an orthodox configuration (Fig. 4L).

## Discussion

Our study examines the disposition of the mitochondrial pool in reprogrammed cells at three phenotypic stages: pre-reprogramming, after forced de-differentiation and assumption of a pluripotent identity, and after re-differentiation to a phenotype similar to the input cell.

The data we report confirms earlier studies that IPSCs display a mitochondrial complement that, in most respects, does not differ significantly from the mitochondrial complement of natural human ESCs. Together, these findings suggest that the nuclear program of cells identifying themselves as “pluripotent” actively promotes a smaller and less active mitochondrial complement — presumably to minimize the potential for DNA damage from ROS — as suggested in the Introduction. Data directly examining ROS and the expression of factors that modulate DNA integrity in IPSCs further confirms this notion [22], [23].

A comparison of mitochondrial morphologies in IPSCs and ESCs using TEM by Prigione et al. [23] found that both cell types had a number of mitochondria with “underdeveloped crista” and an “immature-like morphology characteristic of ESCs”. We observed a similar morphology in IPSC and ESC mitochondria that we interpreted more as a “condensed” configuration than underdevelopment; however, both properties might contribute to the morphological appearance. IPSC/ESCs represent an unusual cellular state – one in which the biological program is prepared for an *in vivo* environment that is oxygen deprived (prior to implantation and establishment of a blood supply), but instead finds itself in the oxygen-rich environment of a cell culture incubator. For this reason, the relationship between mitochondrial structure and function that is well established in somatic cells (see below) may not apply to pluripotent cell types maintained *in vitro*. What is certain, however, is that IPSC and ESC mitochondria are very similar to each other, and all indications are that both cell types have low oxidative phosphorylation and ROS generation, producing in an intracellular environment conducive to the preservation of genomic integrity.

A more pressing question with regard to the practical uses and applications of IPS cells, however, is the relative fitness of the downstream derivatives of IPSCs that can be used to study healthy or diseased human tissues, or, one day, as potential sources for cellular replacement therapy. Although our earlier study documents telomere elongation after reprogramming to pluripotency and resumption of telomere shortening after differentiation, neither this earlier report, nor others published since, provide indications as to whether or not the energetic of the intracellular environment of re-differentiated cells are altered to match the renewal of cellular longevity.

We observed a dramatic increase in cellular respiration in TER cells compared to parental fibroblasts that has not been described previously. This improvement was marked by increases in cellular ATP, mitochondrial membrane potential, and the number of mitochondria of orthodox configuration. We postulate that the compromised mitochondria observed in the FIB cells arose as the result of multiple stressors that accompany advanced cellular age and the approach of senescence — such as ROS damage and increased cell death – that are known to impact mitochondrial configuration and function [27]–[31]. If the condensed and ultracondensed mitochondrial configurations seen in FIB cells are considered indicative of an effort to maximize ATP production [25], [26] as compensation for cellular dysfunction (brought on by the aging process), then mitochondria in the orthodox configuration observed in TER cells may be thought of as a capacitor, fully charged and ready to work in the production of ATP within an environment with sufficient ATP for cellular needs. In short, the likely source of significantly increased membrane potential and ATP production in the TER cells arises from the substantial increase in healthy, orthodox configuration mitochondria with high metabolic capacity.

It is likely that the impact of reprogramming on mitochondria was evident in our experiments and not in two other recent reports [22], [23] because the cells we used carried a relatively compromised mitochondrial complement discussed above. As a consequence, improvements in mitochondrial function that were readily observed in our experiments would probably not be seen in energetically robust cell lines such as those described in other reports.

Another difference in the experimental paradigm that we used was the derivation of differentiated cells from teratoma tissue as opposed to derivation from cells differentiated exclusively *in vitro*. The original objective of this method of obtaining IPSC-derived lines was to push differentiation and replicative cycles to an extreme that would allow us to observe shortened telomeres and the resumption of normal aging to a degree that was measurable by TRF analysis, as previously reported for the TER lines used herein[7]. Another possible explanation for the differences observed between input cells and IPSC-derived cells in this report compared to earlier reports is that TER cells may be more differentiated, more homogenous, and more “fibroblastic” than cells generated by short-term differentiation *in vitro*. Even though cellular morphology and the nuclear program undergo a change after a few population doublings and within the relatively short time-frame of a few weeks, it may be that the mitochondrial complement requires more time and replicative cycles to realize its full potential. Future studies will determine whether extension of *in vitro* differentiation to longer periods will result in mitochondrial improvements similar to those we observed in TER cells.

Another possibility that cannot be ruled out is that the teratoma growth process, which accounts for the vast majority of the replicative cycles the TER lines undergo, also entails a selective process that weeds out energetically inferior cells producing a population of IPSC-derived “super cells”. Regardless of whether the changes we observe between FIB and TER cells is a simple function of input cell robustness, differentiation paradigm, or selection for super cells by many population doublings, our results unequivocally indicate that the derivatives of IPSC lines that have undergone telomere elongation and subsequent resumption of apparently normal aging after differentiation, can possess a mitochondrial complement that displays substantial improvements and indications of cellular rejuvenation in parallel.

It remains to be seen if these results raise new concerns with regard to the eventual use of IPSC derivatives for therapeutic transplants. If *in vitro* methods are not forthcoming, and growth and differentiation within a living animal host is required to “reset” the mitochondria of cells derived from donors with compromised mitochondrial pools (i.e. from age or disease), then it is possible that only IPSCs derived from sources or cell types with robust mitochondrial pools will be suitable to generate good transplant material for some tissue types such as differentiated neurons. On the other hand, these data suggest that IPSC-derived stem cell populations — such as bone marrow, liver, muscle, or skin progenitors – produced from sources with essentially any quality of mitochondria could, over time, improve and maximize their mitochondrial pool following engraftment. In either event, these results suggest that changes in the mitochondria of reprogrammed cells will likely be a consideration of future therapeutic applications.

## Materials and Methods

### Cell Lines

The origin, derivation, and conditions of culture, and characterization of the fibroblast, IPSC, and TER cell lines used in this report have been reported previously [7]. FIBA and FIBB are described therein, IPSCA and IPSCB of this report correspond to line IPSCA3 and IPSCB3, and TERA and TERB correspond to lines TERA3 and TERB3. Line ESC1 in this report is human ESC line H9 and ESC2 is H1 (WiCell Research Institute, Madison, WI). Cell lines were analyzed at passages used in [7], except line ESC2 (H1) which was used at passage 26–28.

### Expression analysis

Phase-contrast imaging, qPCR analysis, and immunocytochemistry was performed were as described [7]. HuNu antibody MAB1281 (Chemicon/Millipore, Billerica, MA) was used at 1∶250, and others as in Ref. 7.

### Measurement of cellular ATP and ADP/ATP ratios

Determination of cellular ATP concentrations was performed using an ATP Bioluminescent Somatic Cell Assay Kit (Sigma, Saint Louis, MO, USA) as previously described[32]. Briefly, duplicate luminometer readings were taken from each sample over 20 s intervals, and the average relative light unit readings were used to determine ATP content in the samples against the standard curve. ADP/ATP ratios were determined using the EnzyLight™ ADP/ATP ratio assay kit from BioAssay Systems (Hayward, CA, USA) according to the manufacturer's instructions.

### Assessment of MMP and Mitochondrial Mass

Evaluation of mitochondrial membrane potential and assessment of mitochondrial mass were performed as previously described [24]. The cell concentration in stained samples was adjusted to 1×10^6^ cells/ml. The cells were stained using the membrane-sensitive dye JC-1 (DePsipher™; R&D Systems, Minneapolis, MN, USA) or nonyl acridine orange (Molecular Probes, Inc., Eugene, OR, USA), and then analyzed by flow cytometry (BD Vantage SE) using a 520-nm filter for the observation of the green fluorescence and 590 nm for the red fluorescence.

### Electron microscopy, tomographic reconstruction and quantification of mitochondrial/crista properties

EM analysis of mitochondria was as previously described[32], [33]. Briefly, three-dimensional reconstructions of portions of the cell containing mitochondria were generated using standard techniques[34]. Sections with a thickness about 500 nm were cut out, and stained for 30 min in 2% aqueous uranyl acetate, followed by 15 min in lead salts. Next, fiducial cues consisting of 15 or 20 nm colloidal gold particles were deposited on either side of each section.

For each reconstruction, a series of images at regular tilt increments were collected with a JEOL 4000EX intermediate-voltage electron microscope operated at 400 kV. Tilt series were recorded at a magnification of 12,000X with an angular increment of 2° from −60° to +60° about an axis perpendicular to the optical axis of the microscope using a computer-controlled goniometer to achieve accurate increments at each angular step. Illumination was held to near parallel beam conditions and optical density maintained constant by varying the exposure time. Images were collected using a slow-scan CCD camera with 4096×4096 pixels at a resolution of 0.71 nm per pixel. The IMOD package[35] was used for generating the reconstructions. Volume segmentation was performed by manual tracing in the planes of highest resolution with the program Xvoxtrace[36], [37]. Mitochondrial reconstructions were visualized using Analyze (Mayo Foundation, Rochester, Minnesota). Segmented and surface-rendered volumes were examined with Synu (National Center for Microscopy and Imaging Research), as described[38]. These programs allow one to step through reconstructed slices in any orientation and to track or model features of interest in three dimensions. Measurements of structural features were made within segmented volumes by the programs, Synuarea and Synuvolume (National Center for Microscopy and Imaging Research). Quantification of the presence of different mitochondrial structures was performed on random transmission EM-acquired images.

Quantification of mitochondrial configuration in each cell line was performed by manual counting of mitochondria of orthodox or condensed configuration in 4–6 representative EM images (of each line) by six unbiased volunteers unfamiliar with the study. The volunteers were instructed as to the visual characteristics of mitochondria of both general configurations at 5800-10000X magnification, and were instructed to count only the mitochondria where the crista disposition — and therefore the configuration — could be definitively visualized for categorization. The results of each count were converted to a ratio of orthodox/condensed mitochondria and the averages and standard errors calculated.

### Statistical analysis

Data were tested for normality using SAS statistical analysis software (Cary, NC). Variables that did not present normal distribution were transformed by log(10) and re-analyzed. Results are presented as untransformed data for ease of understanding. Discrete variables were analyzed by least squares ANOVA, using the GLM procedure of the SAS software with cell type and cell line set as independent variables. The effect of cell line was nested within cell type.
